# Preventive behaviors against COVID‐19 among health care providers in Iran: A cross‐sectional study

**DOI:** 10.1002/hsr2.1839

**Published:** 2024-01-29

**Authors:** Souri Niksirat, Nooshin Rouhani‐Tonekaboni, Maryam Shakiba, Parisa Kasmaei

**Affiliations:** ^1^ Department of Health Education and Promotion, School of Health Guilan University of Medical Sciences Rasht Iran; ^2^ Department of Health Education and Promotion, Research Center of Health and Environment, School of Health Guilan University of Medical Sciences Rasht Iran; ^3^ Department of Biostatistics and Epidemiology, School of Health Guilan University of Medical Sciences Rasht Iran

**Keywords:** behavior, COVID‐19, healthcare providers, prevention, protection motivation theory

## Abstract

**Background and Aim:**

During outbreaks of infectious diseases, if healthcare providers do not follow the principles of prevention, the risk of personal infection increases and they become a source of infection spread. This study aimed to determine the factors related to the preventive health behaviors of COVID‐19 among Iranian healthcare providers based on protection motivation theory (PMT).

**Methods:**

This analytical cross‐sectional study included 346 healthcare providers. Data was collected by an online researcher‐made questionnaire based on PMT. To analyze the data, independent *T* tests, analysis of variance (ANOVA), Spearman correlation coefficient, multiple linear regression, and SPSS 22 software were used. *α* was considered as 0.05.

**Results:**

85.3% of the healthcare providers would always wear masks, 80.7% would always refuse to kiss and touch hands with others, and 34.7% sometimes would exercise at home. The preventive behaviors were significantly correlated with protection motivation (*r* = 0.84), self‐efficacy (*r* = 0.51), response efficiency (*r* = 0.43), perceived severity (*r* = 0.41) Fear (*r* = 0.21), perceived susceptibility (*r* = 0.11), response cost (*r* = −0.14), and reward (*r* = −0.15). PMT constructs were able to predict 77% of the variance of the behaviors and the protection motivation construct was the strongest predictor (*β* = 0.806). Income above 300 Dolars per month was significantly related to the decrease in the mean score of preventive behaviors against COVID‐19. The female sex and the individual or family history of infectious diseases were significantly related to increasing the mean score of COVID‐19 preventive behaviors.

**Conclusion:**

Based on the study results, it is suggested that some educational interventions be designed and implemented with a focus on this construct and the perceived severity construct and that more attention be given to the education of health care providers with high‐income levels, male providers, and the individuals without a history of corona infection in themselves or their family members.

## 1 INTRODUCTION

Preventing and controlling the spread of infectious diseases, especially infectious diseases due to the high speed of spread and widespread consequences such as the death of children, adolescents, and adults are considered critical issues in the community's health.^1^, ^2^ Also, the ongoing COVID‐19 pandemic has strengthened views on the economic and social consequences of infectious diseases; and the closure of some businesses and warnings of continuous stay at home have been followed by psychological consequences such as depression and anxiety.^3^, ^4^

In outbreaks of infectious diseases, healthcare providers, as the first line of healthcare delivery, are more exposed to infection than others.^5^ During the corona pandemic, the risk of infection and involvement of health workers with this disease increased because the control of the coronavirus in the community required identification processes, treatment of infected cases, isolation of infected cases, and tracking and quarantine of people in close contact with patients.^5^, ^6^ Therefore, the implementation of preventive measures taken by healthcare providers to protect themselves from infection is one of the most essential strategies for the prevention and control of viral respiratory infections,^7^ because healthcare providers, on the one hand, are directly in contact with infected patients and on the other hand in direct contact with healthy people. Hence, ensuring the safety of these workers not only protects them from infection but also prevents the spread of infection in the area and the entire community.^8^, ^9^

Although the Covid‐19 crisis has led to an increase in personal protective equipment among nurses, almost 20% of these employees do not use these protective equipment.^10^ Therefore, identifying the determining and contributing factors in using personal protective equipment and implementing preventive measures and control of viral diseases is of particular importance.^11^

One of the ways to encourage employees and change their behavior by implementing protective measures is health education. To increase the effectiveness of training, the first step is to choose the appropriate model. In this case, the program is launched in the right position and moves in the right direction.^12^

The PMT is one of the educational theories in health education which is used to change health‐related behaviors. This theory is based on the value expectancy theory which has been used to investigate the factors affecting motivation and individual health behavior.^13^, ^14^

According to Rogers, fear affects protection motivation or intention through five constructs to behave protectively against health risk and the motivation for protection ultimately stimulates health behavior. These five constructs include: (1) self‐efficacy (a person's belief that she/he can perform protective behaviors successfully), (2) perceived response efficiency (a person's expectation that a consistent response, which is a protective behavior against health risk, can eliminate the risk), (3) perceived susceptibility (a person's belief that she/he is vulnerable to a health risk), (4) perceived severity (a person's belief that the risk is serious), (5) perceived response cost (a person's estimate of the costs related to his/her protective behavior such as money, person, time, effort).^15^

Considering that COVID‐19 is a threat to human life and human communities will face similar conditions in the future, also considering the important role of using PMT assumptions to discover and explain preventive health behaviors, especially in infectious diseases and specifically COVID‐19,^16^, ^17^, ^18^, ^19^, ^20^, ^21^ and since no study based on this theory has been done on health workers in the country, and on the other hand, the role of this group in providing services, transmission of infection, and culture creating for implementation of preventive behaviors in communities, this study aimed to investigate factors related to preventive health behaviors of COVID‐19 based on PMT among health care providers in Guilan Province, Iran.

## 2 MATERIALS AND METHODS

### 2.1 Type of study, sample size, and sampling

The present analytical cross‐sectional study was included healthcare providers working for comprehensive healthcare centers in Guilan Province. Based on Ezati Rad et al.^22^ and Limkunakul et al.^23^ resuls and according to Cochran's formula, *p* = 0.7, *q* = 0.3, and *d* = 0.05, the initial sample size was considered to be 323 people, and considering the 10% probability of dropping out, the final sample size was estimated to be 355 people. healthcare providers working for comprehensive healthcare centers in Guilan Province were included. Sampling was performed using multistage cluster random sampling. Initially, each of the five geographical regions of Guilan Province (e.g., north, south, center, east, and west) was considered a cluster. Then, one to two cities were randomly selected from each geographical region. The selected cities were Somesara, Shaft, Anzali, Astara, Rudbar, Rasht, Lahijan, and Langarud. Lastly, sampling was performed for each city proportionately based on the number of health providers.

### 2.2 Inclusion and exclusion criteria

All the employees who agreed to participate in the study were included in the study, and the employees who had taken leave for more than 4 months at the time of the study and incomplete questionnaires were excluded from the study.

### 2.3 Study implementation

Due to the Corona situation, the online questionnaire was designed in the online Porsline questionnaire software and the form builder and its link was provided to the healthcare workers in comprehensive health centers through SMS. In the desired link, after studying the objectives of the study and being aware of the confidentiality of their information, the participants completed the questionnaire if they wished. The duration of completing the questionnaire was 10–15 min.

### 2.4 Instrument

Data were collected using a questionnaire consisting of three sections: demographic characteristics, the PMT constructs and preventive health behaviors.

Demographic information included age, gender, education, occupation, marital status, place of residence, monthly income, health status (subjective), underlying diseases, history of corona disease in the person, relatives and acquaintances. The PMT constructs included 8 dimensions: motivation of protection (14 questions) for example, “I'm going to keep wearing masks at work or crowded places by the end of the coronavirus epidemic,” Perceived susceptibility (4 questions) such as “I am not likely to be infected with corona,” perceived severity (4 questions) for example “corona disease can lead to death,” Perceived response efficacy (9 questions) for example “Preventive behaviors reduces the risk of coronavirus,” Perceived response cost (5 questions) for example “Mask is hard to use,” reward (5 questions) such as “I breathe comfortably when I don't wear a mask,” fear (4 questions) for example “Catching the coronavirus and its complications scares me,” and perceived self‐efficacy (9 questions) for example “Doing exercise to prevent the spread of corona is hard for me.” Also, preventive health behaviors with 14 items were included for evaluation included: wearing mask, hand washing regularly, keeping social distance, not attending crowded places, not going on trips, parties, celebrations and mourning, using disinfectant gel or solution, healthy eating habits, and having physical activity.

Perceived susceptibility, perceived intensity, response efficiency, response cost, reward and fear were rated on a 5‐point Likert scale from 1 (*strongly disagree*) to 5 (*I strongly agree*), self‐efficacy and protection motivation on a 5‐point Likert scale from 1 (*not at all*) to 5 (*very high*) and finally preventive health behaviors on a 5‐point Likert scale from 1 (*never*) to 5 (*always*).

The data collection tool was a researcher‐made questionnaire inspired by similar studies conducted by Bashirian et al.^18^ and Farooq et al.^21^ After holding several sessions with the research team, the initial questionnaire was revised and changed. The content validity of the questionnaire was confirmed by a specialized panel consisting of nine faculty members (seven health education and health promotion specialists, one epidemiologist and one statistics specialist). After removing some questions and corrections, the mean values of CVR and CVI respectively regarding the structure's perceived sensitivity 1 and 1, perceived severity 0.94 and 1, response efficiency 1 and 1, perceived self‐efficacy 1 and 1, response cost 0.95 and 1,1, rewards 1 and fear 1 and protection motivation 0.98 and 1 and preventive behaviors 0.98 and 1 were obtained.

According to Lawshe Table including nine specialists, the minimum desired score was 0.78, which was approved for all the items. To determine the reliability of the instrument, the questionnaire was completed by 20 healthcare providers outside the study population. The internal consistency of the constructs based on Cronbach's alpha and the intraclass correlation coefficient was calculated using test‐retest (10‐day interval). Cronbach's *α* coefficient was found 0.75 for perceived susceptibility, 0.65 for perceived intensity, 0.80 for response efficiency, 0.77 for perceived self‐efficacy, 0.71 for response cost, 0.71 for rewards, 0.71 for fear, 0.82 for protection motivation, and 0.73 for preventive behaviors.

### 2.5 Data analysis

Data were described using mean and standard deviation for quantitative variables and absolute and relative frequency for qualitative variables. The normality of the data was measured using Kolmogorov and Smirnov tests and skewness and elongation indices. Independent *T* tests and analysis of variance were used to compare the scores of preventive behaviors. The Spearman correlation coefficient was used to determine the correlation coefficient between the variables in question, and multiple linear regression (MLR) was also used to investigate the predictive relationship between the variables and the preventive behavior. The significance level was less than 0.035. Data analysis was performed using SPSS software version 22 at *α* = 0.05 significance level.

### 2.6 Ethical consideration

Before collecting the data, first, a research license from the Ethics Committee of Guilan University of Medical Sciences was obtained with the code number IR. GUMS. REC.1400.007. Before the conduct of the study, an informed consent form was also completed by the subjects as well. Needless to say, the purpose of the study was briefly explained to the subjects in the questionnaire guide.

## 3 RESULTS

Data of 346 participants were entered in the analysis. Fifty‐seven percent of participants had 40 to 60‐year‐old; 86.1% were women, 63.9% were single and 76.6% were married. The majority of them (93.1%) lived in the city and their income (44.5%) was between 300 and 450 dolars. 60.1% described their health status as good; 62.1% of them did not have a systemic disease; and 40.46% including themselves and/or their relatives, had already been exposed to coronavirus. 85.3% of the health care providers would always wear masks, 80.7% would always refuse to kiss and touch hands with others, and also 34.7% sometimes would exercise at home. More details were presented in Table 1.

**Table 1:** Demographic characteristics of the studied health care workers (*n* = 346).

Characteristics		Number	Percent
Age	20–30 years	66	19.1
	30–40 years	83	24
	40–50 years	148	42.8
	50–60 years	49	14.2
Sex	Female	298	86.1
	Man	48	13.9
Educational level	Diploma	40	11.6
	Associate	33	9.5
	Bachelors	221	63.9
	Masters	52	15
Employment status	Official	193	8.55
	Contractual	99	28.6
	corporate	9	6.2
	Manpower plan	45	13
Marital status	Single	75	21.7
	Married	265	76.6
	Divorced and widow	6	1.7
Residence	City	322	93.1
	Edge of the city	6	1.7
	Village	18	5.2
Monthly income (dollars)	<150	12	3.5
	150–299	65	18.8
	300–450	154	44.5
	>450	115	33.2
Health status (subjective)	Excellent	60	17.3
	Well	208	60.1
	Medium	75	21.7
	Weak	3	0.9
Disease	Diabetes	12	3.5
	High blood pressure	29	8.4
	Cardio‐vascular	5	1.4
	Respiratory	12	3.5
	Specific diseases	4	1.2
	Another	69	19.9
	None	215	62.1
History of corona disease in person, relatives and acquaintances	Person and relatives	140	40.46
	Person	18	5.20
	Relatives	123	35.55
	None	65	18.79

Table 2 shows the descriptive indicators of the PMT constructs as well as the score of corona infection preventive behaviors and the correlation between the constructs. According to Table 2, the average percentage of the maximum score was achieved at a high level for perceived severity, perceived response efficacy, protection motivation, behavior, fear, perceived susceptibility, and perceived self‐efficacy, whereas it was found to be moderate for perceived response cost and rewards. Moreover, 85.3% of the health care providers would always wear masks, 80.7% would always refuse to kiss and touch hands with others, and also 34.7% sometimes would exercise at home.

**Table 2:** Mean, standard deviation, average percentage of the maximum obtainable score, the lowest and the highest score obtained and the matrix of Spearman's correlation coefficient between the constructs of protection motivation theory and preventive behaviors against corona.

Variable	Mean (standard deviation)	The average percentage of the maximum obtainable score	The lowest and highest score obtained	1	2	3	4	5	6	7	8	9
Motivation of protection	61.14 (6.56)	84.17	43–70	0.84	1							
Perceived susceptibilities	16.50 (2.69)	78.12	4–20	0.11	0.09	1						
Perceived severity	18.43 (1.83)	90.18	10–20	0.41	0.41	0.50	1					
Perceived response efficacy	39.96 (3.92)	86	29–45	0.43	0.44	0.21	0.44	1				
Perceived response cost	15.20 (4.26)	51	5–25	−0.14	−0.15	0.03	−0.02	−0.25	1			
Reward	16.71 (4.03)	58.55	5–25	−0.15	−0.17	0.01	0.003	−0.19	0.51	1		
Fear	16.93 (2.88)	80.81	7–20	0.21	0.28	0.31	0.45	0.26	0.19	0.14	1	
Perceived self‐efficacy	36.31 (4.21)	75.86	25–45	0.51	0.54	0.10	0.38	0.46	−0.32	0.25	0.15	1
Preventive behaviors	60.22 (5.633)	82.53	40–70	1								

According to the results, there was a significant correlation between the constructs of PMT and coronavirus preventive behaviors. More particularly, there was a high positive correlation for protection motivation (*r* = 0.84), a moderate positive correlation for self‐efficacy (*r* = 0.51), perceived response efficacy (*r* = 0.43) and perceived severity (*r* = 0.41), and a low positive correlation for fear (*r* = 0.21) and perceived susceptibility (*r* = 0.11). This means that these factors with different intensities strengthen the adoption of preventive behaviors against COVID‐19 by people.

However, there was a low inverse (negative) correlation for perceived response cost (*r* = −0.14) and a low inverse (negative) correlation for reward (*r* = −0.15). This means that these two factors are an obstacle for people to adopt preventive behaviors against COVID‐19.

Table 3 shows a final model with the forward approach of predicting the protection motivation construct based on the background variables and the PMT constructs. The self‐efficacy construct had the highest coefficient of determination, which by itself would predict 34% of the variance of protection motivation. This construct was also the most important predictive variable in the model as the standardized coefficient was 0.41. By increasing one point in the self‐efficacy score, the mean protection motivation score of 0.65 showed a significant increase. Likewise, the response efficiency construct (standardized coefficient = 0.21) together with the self‐efficacy construct showed the possibility to predict 39% of the variance of the protection motivation construct. The perceived severity construct with a standard coefficient of 0.14 was in the next rank in terms of the predictive power of protection motivation variance. The mean score of protection motivation in individuals with no history of infectious diseases in themselves or others was significantly higher (1.88 points) than in those with a history of infectious diseases in themselves and others. The determination coefficient of the final model was also measured at 42%.

**Table 3:** Prediction of protection motivation based on the theory of protection motivation using multivariable linear regression model with forward approach.

Variables	*B*	SE	Beta	*t* Value	*p* Value	Confidence interval 95%	
						Upper	Under
Self‐efficacy	0.646	0.076	0.414	8.47	0.001	0.769	0.495
Response efficiency	0.346	0.085	0.205	4.07	0.001	0.179	0.513
Perceived severity	0.500	0.174	0.140	2.87	0.004	0.157	0.843
*History of corona disease*							
Person and relatives	Reference						
Person	0.723	1.288	0.024	0.56	0.575	3.258	−1.812
Relatives	0.390	0.655	0.028	0.60	0.552	1.680	−0.899
None	1.83	0.817	0.105	2.30	0.022	3.491	0.274
*Health status*							
Weak	Reference						
Medium	0.559	3.022	0.034	0.18	0.853	6.504	−5.386
Well	−1.141	2.986	−0.085	−0.38	0.703	4.733	−7.016
Excellent	−0.812	3.044	−0.047	−0.27	0.790	5.177	−6.801
(Constant)	14.819	4.618		3.21	0.001	23.905	5.732

According to Table 4, the final model with the forward approach of predicting the protection motivation construct based on the background variables and the PMT constructs indicates the possibility to predict 77% of the changes in individuals' preventive behaviors. The protection motivation construct had the highest coefficient of determination; it would by itself predict 72% of the variance of behavior and was the most important predictive variable in the model (standardized coefficient: 0.81). Perceived severity showed a significant relationship with COVID‐19 preventive behaviors (*B* = 0.22). The results showed that the average score of preventive behaviors against covid‐19 in people with a monthly income of fewer than 300 dolars is significantly higher than in people with a monthly income of more than 300 dolars (*p* = 0.003) and the average score of preventive behaviors against covid‐19 It was significantly more in women than in men (*p* = 0.001). Also, the average score of preventive behavior in people who had a personal experience of being infected with corona or one of their family members was infected with covid‐19 was 1.12 points higher than people who had no personal or family history of being infected with covid‐19 and the difference was statistically significant (*p* = 0.012).

**Table 4:** Predicting behaviors to prevent corona infection based on protection motivation theory using multivariable linear regression model with forward approach.

Variables	*B*	SE	Beta	*t* Value	*p* Value	Confidence interval 95%	
						Upper	Under
Protection motivation	0.690	0.025	0.806	27.59	**0.001**	0.739	0.641
*Monthly income (dolars)*							
<150	Reference						
150–299	−0.796	0.905	−0.055	−0.88	0.380	0.984	−2.576
300–450	−1.938	0.879	−0.170	−2.21	**0.028**	−0.209	−3.668
>450	−2.645	0.876	−0.221	−3.02	**0.003**	−0.921	−4.369
*Sex*							
Female	Reference						
Male	−1.965	0.438	−0.121	−4.48	**0.001**	−1.102	−2.828
Perceived severity	0.225	0.090	0.073	2.48	**0.013**	0.403	0.046
*History of corona disease*							
Person and relatives	Reference						
Person	1.245	0.695	0.049	1.79	0.074	2.615	−0.123
Relatives	0.454	0.347	0.038	1.31	0.193	1.138	−0.230
None	1.118	0.442	0.073	2.53	**0.012**	1.988	0.247
(Constant)	17.531	2.16		8.11	**0.001**	21.787	13.276

*Note*: Bold values are significant *p* < 0.05.

## 4 DISCUSSION

In this study, which investigated the factors related to the preventive health behaviors of COVID‐19 among Iranian healthcare providers based on PMT, was found that 82.35% of the subjects would perform preventive behaviors of COVID‐19 including wearing mask, hand washing regularly, keeping social distance, not attending crowded places, not going on trips, parties, celebrations and mourning, using disinfectant gel or solution, healthy eating habits, and having physical activity. In a study conducted by Azadeh and colleagues, protective behaviors against COVID‐19 were reported at 69.8%.^24^ Similar findings were also reported in Nasirzadeh and colleagues study (88.42%).^25^ In another study, Ashrafi et al.^26^ observed the skills of the groups at an average level at the beginning of their investigation. One of the reasons for these factors being high in the present study relates to the difference in the type of job between the studied groups because the employees of comprehensive health services centers were exposed to patients infected with corona and therefore had higher awareness than other people in society. In other words, high awareness seems to be effective in adopting preventive behaviors.^25^, ^27^

Performing the preventive behaviors of COVID‐19, in Iranian health providers was lower than other countries. In Saudi Arabia, the vast majority 85.7% of HCPs answered “always” regarding the behavioral intention of health care providers to comply with COVID‐19 preventive behavior.^28^ In Thailand, the preventive behavior category had an average score of 87.6%.^23^

According to the results, there was a significant correlation between the PMT constructs and COVID‐19 preventive behaviors and protection motivation showed a strong positive correlation with preventive behaviors. In two other studies carried out by Azadeh et al.^24^ and Ashrafi et al.^26^ a positive and significant relationship was found between protection motivation and protective behavior, which is in line with the results of the present study. It seems that increasing protection motivation to facilitate protective behaviors can be considered a noteworthy principle in education.

In the present study, perceived self‐efficacy, response efficiency and severity had a moderate positive correlation with preventive behaviors, which is consistent with the findings offered by Azadeh et al.^24^ and Bashirian et al.^18^ In another study conducted by Ashouri‐Ahmadgourabi et al.^29^ self‐efficacy was found to have a similar correlation with behavior. Self‐efficacy plays an important role in changing behavior, and individuals with the most behavior changes have a higher level of self‐efficacy at the outset.^30^

In the present study, the construct of perceived sensitivity (*r* = 0.11) also had a very low positive correlation with behavior. In terms of assessing the risk perception of COVID‐19 among medical students, Taghrir and colleagues found a significant relationship between risk perception and health behaviors.^31^ In the present study, there was also a low positive correlation between the construct of perceived fear of disease risks and protective behaviors. In another study, Zare and colleagues reported the construct of perceived fear of disease risks as the weakest predictor for protective behaviors.^32^ In the present study, it was found that the healthcare providers' awareness,susceptibility and fear of the consequences of the disease would cause them to show preventive behaviors. In addition, there was a great amount of susceptibility and fear of disease as well as preventive behavior in the studied population, while the correlation found between these constructs and behavior was rather different. Therefore, it seems that there are some other effective factors involved in the individuals' protective behaviors during the COVID‐19 pandemic, and more research is obviously needed in this regard.

In a previous study in the literature, there was also a very low negative correlation reported between the perceived response cost and the reward and preventive behaviors.^33^ In other words, the high perceived cost of healthy behaviors concerning COVID‐19 would make individuals less motivated to behave protectively. Based on the results of this study, however, self‐efficacy was found to be the most predictive construct of protection motivation among the constructs of PMT; this construct had a positive effect on the individuals' willingness to protect themselves against the disease. More particularly, the highest coefficient of determination was related to self‐efficacy, which would predict 34% of the variance of behavior intention. Reaching the standardized coefficient of 0.41, this construct was the most important predictive variable in the model, which was consistent with the results of the study conducted by Azadeh and colleagues who considered self‐efficacy an effective factor in employees' willingness to protect themselves against the disease.^24^ In another study, self‐efficacy was reported as the most important prerequisite and the strongest predictor of protection motivation.^34^

In predicting protective behaviors through the PMT constructs, the highest coefficient of determination was related to the protection motivation construct, which would predict 72% of the variance of behavior. With a standardized coefficient of 0.81, protection motivation was the most important predictive variable in the model. Results of some other studies are similarly in line with the present study.^35^, ^36^

In this regard, a similar study showed that protection motivation was a factor affecting the protective behaviors of COVID‐19.^18^ Ashrafi and colleagues observed a positive and significant relationship between intention and protective behavior.^20^, ^26^ It seems that adopting recommended health behavior (protective behavior) against health risks is a direct act of motivation for self‐protection.^28^ Also, perceived severity predicted 7.3% of behavior variance. According to the assumptions of PMT, when individual believes that the risk is serious and is convinced, occurs behavior change.^37^

According to the findings, demographic factors such as the female sex, having an income of fewer than 300 dolars and no history of coronavirus would also predict preventive behaviors. It is safe to conduct that individuals with low‐income levels would consider themselves vulnerable to the disease, and females were more responsible for their health and of course, their family health and lack of immunity to disease in individuals without a history of corona infection would cause them to adopt preventive behaviors more seriously. In this regard, previous studies also showed gender, economic status, education, and age as predictive variables.^38^, ^39^, ^40^, ^41^

According to the conditions created in the COVID‐19 crisis, in most of the studies, according to what was done in the present study, questioning has been done online. All studies were cross‐sectional and were conducted to assess current behavior. In intervention studies, the first step is to compile educational content based on the desired educational theory.^42^ In the field of infectious such as HIV, the implementation of face‐to‐face programs seems to be more effective,^42^ but the experience of the COVID‐19 crisis showed that in the case of diseases with a high transmission rate, the implementation of online training programs has a higher ability to be implemented.

One of the strengths of the study was the assessment of health care providers which are role models of clients to comprehensive health service centers and they play a major role in the sustainability of individuals' health behaviors. Another strength of the study was the high extent of the studied population, which makes it easy to generalize the results. A wide range of preventive behaviors was investigated, which can have a major effect on conducting future educational interventions.

Like all studies, the present study had some limitations. The most important limitation of this online study was the self‐reporting of the questionnaire, which should be done with caution when generalizing the results to the general public.

## 5 CONCLUSION

Given the predictive power of PMT and the key role of the protection motivation construct in predicting preventive behaviors, it is suggested that some educational interventions be designed and implemented with a focus on this construct and the perceived severity construct and that more attention be given to the education of health care providers with high‐income levels, male healthcare providers, and the individuals with a history of corona infection in themselves or their family members.

## AUTHOR CONTRIBUTIONS

**Souri Niksirat**: Conceptualization; data curation; investigation; methodology; writing—original draft; writing—review & editing. **Nooshin Rouhani‐Tonekaboni**: Conceptualization; data curation; funding acquisition; investigation; methodology; project administration; resources; writing—original draft; writing—review & editing. **Maryam Shakiba**: Conceptualization; data curation; formal analysis; methodology; project administration; writing—original draft; writing—review & editing. **Parisa Kasmaei**: Conceptualization; data curation; methodology; resources; writing—original draft; writing—review & editing.

## CONFLICT OF INTEREST STATEMENT

The authors declare no conflict of interest.

## TRANSPARENCY STATEMENT

The lead author Nooshin Rouhani‐Tonekaboni affirms that this manuscript is an honest, accurate, and transparent account of the study being reported; that no important aspects of the study have been omitted; and that any discrepancies from the study as planned (and, if relevant, registered) have been explained.

## Data Availability

The data set is not publicly available. Requests to access these data sets should be directed to the corresponding author.
